# Design Method of Lightweight Metamaterials with Arbitrary Poisson’s Ratio

**DOI:** 10.3390/ma11091574

**Published:** 2018-09-01

**Authors:** Haoxing Qin, Deqing Yang, Chenhui Ren

**Affiliations:** State Key Laboratory of Ocean Engineering, Collaborative Innovation Center for Advanced Ship and Deep-Sea Exploration, School of Naval Architecture, Ocean and Civil Engineering, Shanghai Jiao Tong University, Shanghai 200240, China; qinhaoxing@163.com (H.Q.); renchenhuicn@sjtu.edu.cn (C.R.)

**Keywords:** lightweight, metamaterials, functional element, topology optimization, deformation resistance, vibration reduction

## Abstract

A heuristic approach to design lightweight metamaterials with novel configurations and arbitrary Poisson’s ratio is studied by using the functional element topology optimization (FETO) method. Mathematical model of the optimization problem is established, where the minimization of the mass is set as the objective, then a series of metamaterials with Poisson’s ratio ranging from −1.0 to +1.0 are designed by solving this model. The deformation resistance and vibration reduction performance of the novel metamaterials and conventional honeycomb are compared by numerical simulations. Specific stiffness analysis shows that the novel metamaterials are 5.6 to 21.0 times more resistant to deformation than that of the honeycomb, and frequency response shows about 60% improvement in vibration reduction performance. Finally, the lightweight effects of the novel metamaterials on deformation resistance and vibration reduction performance are analyzed, and further analysis reflects that the lightweight effects increase with the increase of the absolute value of the Poisson’s ratio.

## 1. Introduction

The unusual properties of metamaterials have been proven to add significant improvement compared to some traditional materials, especially regarding their mechanical properties related to Poisson’s ratio (PR), such as impact resistance [1,2], weight [3], energy absorption [4,5], etc. Metamaterials with improved properties have demonstrated their efficiency in several practical fields, such as defense, transportation and aerospace [6,7]. Moreover, the successful manufacturing of metamaterials in recent years [8,9] has also made these unusual features highly valued by materials scientists and physicists [10].

Previous studies on lightweight metamaterials have focused more on evaluating their mechanical properties through simulations and experimental tests, which are not suitable for the application in specific aspects [11]; however there is a lack of systematic methodology for designing novel configurations. It is well known that the mechanical properties of metamaterials are determined by special design and periodic arrangement of the substructure configurations; thus, the internal configurations of substructures play a decisive role in the extraordinary properties, and many interesting properties can be obtained by designing novel substructures [12,13].

The adoption of an optimization design method makes it convenient to design metamaterials with specific properties [14,15]. In two of the common optimization methods, size and shape optimization are characterized by convenience and practicality [16], where metamaterials are designed by optimizing cell wall thickness, cell angle, cell height, and cell length [17,18]. To summarize, these optimizations are still based on the known configurations of metamaterials; consequently, they cannot improve the mechanical properties to a greater extent than changing topology configurations [19,20]. For these reasons, this work introduces a higher-level topology optimization method, which provides a broader margin for designing novel topological configurations of metamaterials. The advantage of topology optimization over the former two methods (size and shape optimization) is that the optimization calculation will automatically find the optimal structure in the design domain instead of locally optimizing the pre-specified configurations of metamaterials [21].

Topology optimization design methods of metamaterials can be classified from microscopic to macroscopic scales. On one hand, microscopic topology optimization [22] focuses on the design of the microscopic materials’ layout, instead of paying attention to the distribution of materials on a macroscopic path. On the other hand, concurrent topology optimization [23] achieves both microscopic materials design and macroscopic structural design. Nonetheless, mechanical properties of metamaterials are mainly determined by the special designs of macroscopic substructure configurations and the periodic arrangement of them, rather than the properties of the constituent materials [11,12]. Consequently, previous work by Qin et al. [24] proposed a macroscopic design method of functional element topology optimization (FETO) that only performed topology optimization design for the macroscopic substructures. The general idea of the FETO method can be depicted as metamaterials comprised of periodically arranged optimal substructures that are designed by topology optimization. Obviously, this method can not only improve the mechanical properties effectively, but also promote the calculation efficiency. Thus, the method of FETO is introduced into this work for designing metamaterials with novel configurations.

Moreover, metamaterials with lightweight properties are being widely used in the applications for structural weight reduction, ranging from hexagonal honeycombs to disordered 3D skeletal networks of foams and sponges [25,26]. In this work, weight minimization is chosen as the goal of an optimization problem to design a series of lightweight metamaterials with arbitrary PR.

Most materials present positive PR effects and tend to get thinner when stretched, whereas negative PR materials are laterally expanded, showing an abnormal “swelling” behavior [27]. Previous work by Carta et al. [28] shows that the PR directly affects the mechanical properties of materials, such as indentation resistance, shear modulus, impact absorption and damage tolerance [29,30]. Therefore, we design a series of metamaterials with various negative and positive PRs to study the influence of PR values on mechanical properties.

This work is organized as follows. Section 2 begins with the introduction of the FETO method and summarizes the measuring method of macroscopic PR effects during the iteration of topology optimization. In Section 3, a mathematical model of the optimization problem is established and solved to design lightweight metamaterials. Section 4 and Section 5 analyze the deformation resistance and vibration reduction performance of these metamaterials, respectively. Section 6 analyzes the lightweight effects on deformation resistance and vibration reduction.

## 2. Functional Element Topology Optimization Method 

This section describes the FETO design method in detail, and the calculation of the macroscopic PR effect is introduced to evaluate the equivalent PR of the functional element during the iteration of optimization.

### 2.1. Definition of Functional Elements and Metamaterials

**Figure 1:**
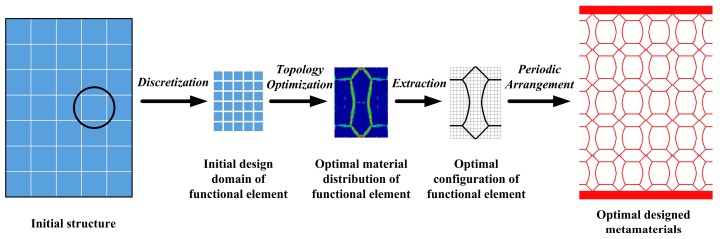
Schematic of metamaterials design method based on functional element topology optimization (FETO) method.

Figure 1 illustrates the main idea of designing metamaterials via the FETO method. A metamaterial to be designed is divided into a certain number of macroscopic substructure domains, and each domain is defined as a Functional Element. Then the single functional element is discretized into a fine finite element mesh and used for structural topology optimization. That is, the essence of FETO is to seek optimal material distribution in the domain of a functional element.

### 2.2. Macroscopic Poisson’s Ratio Effect of Functional Elements

Poisson’s ratio (PR) is defined as the negative ratio of the transverse contraction strain to the longitudinal extension strain with respect to the direction of the stretching force applied, as tensile deformation is considered positive and compressive deformation negative [31]. As a key parameter of metamaterials [28], the evaluation of PR values for metamaterials with common cell configurations follows specific calculation methods [11,32,33], and these accepted PR evaluation methods are based on deterministic configurations [34]. However, the general calculation method of PR for novel configurations is still lacking. In addition, structural uncertainty and the existence of large numbers of irregular holes during the optimization iteration are not conducive to the calculation of PR. For this reason, we introduce an evaluation of macroscopic PR effects to solve these two problems. 

**Figure 2:**
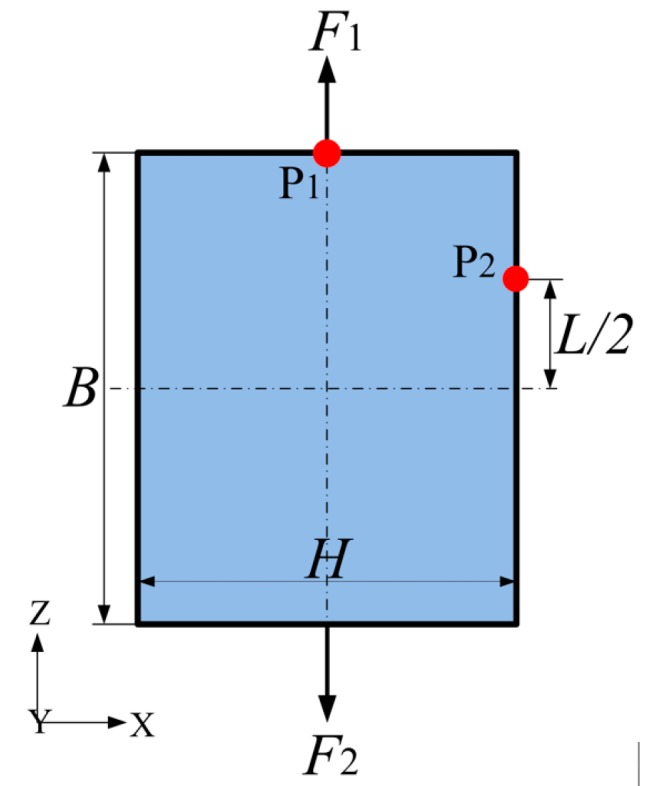
Initial design domain of a functional element.

The work by Schwerdtfeger and Guan et al. [35,36] provides a heuristic idea to study the macroscopic mechanical properties of materials by applying simple loads, which lays the foundation for the macroscopic characterization of mechanical properties of materials in complex stress states. In addition, Carneiro et al. [37] studied the PR by numerical simulation in which the cell of cellular materials is regarded as a macroscopic material and the PR is defined according to the PR test in material mechanics, where the PR is expressed as a negative ratio of the strain in the orthogonal load direction to the strain in the load direction. In view of the validity of the PR evaluation described above, the method by Carneiro et al. [37] is also used to describe the macroscopic PR effect during the iteration of topology optimization of a functional element. As shown in Figure 2, when the element is stretched along the Z-direction, the points P_1_ and P_2_ move in the Z-direction and X-direction, respectively, and the strain of the cell configuration is:

(1) $$\varepsilon_{X}=2\frac{\Delta x_{2}}{x_{2}},\varepsilon_{Z}=2\frac{\Delta z_{1}}{z_{1}}$$

where $\varepsilon_{X}$ is the tensile strain in the stretching direction and $\varepsilon_{Z}$ is the tensile strain perpendicular to the applied load. $\Delta x_{2}$ and $x_{2}$ are the displacement and coordinate position of point P_2_ in the X-direction, respectively. $\Delta z_{1}$ and $z_{1}$ are the displacement and coordinate position of point P_1_ in the Z-direction, respectively. Then the macroscopic PR value can be described as:

(2) $$\nu_{ZX}=-\frac{\varepsilon_{X}}{\varepsilon_{Z}}$$

Figure 2 shows the initial design domain and loading conditions of the optimization design problem, with domain dimensions of *B* = 42 mm, *H* = 30 mm and *L* = 26 mm. The design domain is divided into 84 × 84 elements of type PSHELL. The upper and lower boundaries are simultaneously bearing the vertical tensile loads. Two points are chosen to measure the macroscopic PR effect, corresponding to points P_1_ and P_2_ in Figure 2. The constitutive materials are isotropic and the properties of density, Young’s modulus, and material PR are ρ = 1180 kg/m^3^, *E*_0_ = 2636 MPa, and $\nu_{s}$ = 0.38, respectively. 

According to the definition of PR in Equation (2), the macroscopic PR can be expressed as:

(3) $$\nu =-\frac{\varepsilon_{2}}{\varepsilon_{1}}=-\frac{\Delta u_{2}}{\Delta w_{1}}\cdot \frac{L}{H}$$

where the Z-direction displacement of point P_1_ is denoted by $\Delta w_{1}$, and the X-direction displacement of point P_2_ is denoted by $\Delta u_{2}$.

## 3. Design Method of Lightweight Metamaterials

### 3.1. Topology Optimization Theory Used in FETO Method

Briefly, topology optimization retains the mesh elements that are favorable to the structural force path and remove the mesh elements that have little effect on the configuration through iterative calculations. Topology optimization is now a well-established field. Indeed, numerous topology optimization methods, such as the homogenization method [38], solid isotropic materials with penalization (SIMP) [39], evolutionary structural optimization (ESO) [40], the level set method [41], etc., now exist. In particular, SIMP uses the so-called “power-law approach”, with a wide range of engineering applications; the general idea is to introduce a fictitious density variables field to penalize, for each element, some relevant physical quantities like element stiffness tensor, material density, etc. The value of the pseudo-density at each element centroid is taken as a design variable and the optimum value is provided at the end of the optimization process. In this way, the topology optimization problem is transformed into a classical parametric optimization problem.

In this work, topology optimization is performed with commercial software Hyperworks/OptiStruct (Altair HyperWorks 13.0; Altair Engineering Inc.; Troy, MI, USA), and the SIMP method is implemented in a finite element formulation in OptiStruct. The mathematical expression of SIMP is:

(4) $$E\left(x_{e}\right)=E_{\min}+x_{e}^{p}\left(E_{0}-E_{\min}\right)$$

(5) $$K=\sum_{e=1\;}^{N}\left(E_{\min}+x_{e}^{p}\Delta E\right)\cdot k_{0}$$

where $x_{e}\left(e=1,2,\cdots ,N\right)$ is the material’s relative density of *e*-th element, and *N* is the number of elements. $E_{0}$ and $E_{\min}$ are the initial elastic modulus of the elements and the elastic modulus of the hole elements, respectively; $\Delta E=E_{0}-E_{\min}$, $E_{\min}=E_{0}/1000$. p is the penalization power. $E\left(x_{e}\right)$ is the elastic modulus after interpolation. K is the global stiffness matrix, which can be obtained as the sum of elemental stiffness over all *N* elements, and $k_{0}$ is the initial stiffness matrix of each mesh element.

Since $E_{\min}\ll E_{0}$, Equation (4) can be reduced to:

(6) $$E\left(x_{e}\right)=E_{\min}+x_{e}^{p}E_{0}$$

### 3.2. Mathematical Model of Metamaterials Lightweight Design 

It should be noted that the common optimization objective functions include maximum compliance, minimum compliance and minimum mass. The mathematical model with minimum compliance as the objective function is suitable for designing optimization problems with maximum load bearing performance; that is the greater the bearing stiffness of the structure, the smaller the compliance value. A mathematical model with minimum compliance as the objective function can be used to design a structure with optimal characteristics of energy absorption. In addition, the optimization problem with minimum mass as the objective function enables the design of lightweight materials or structures. These three types of optimization design problem are studied in detail in the work by [24].

In this section, the goal of minimizing mass is used to design metamaterials with lightweight properties. Three components of the topology optimization model are, under the premise of limited amount of materials: PR value as the constraint, relative density of materials as the design variable, and minimization of mass as the objective. Then, metamaterials with various Poisson’s ratios are designed to reveal the effect of PR value on vibration reduction and energy absorption. The mathematical expression of the topology optimization problem is as follows:

(7) $$\mathrm{find}\;X={\left\{x_{1},x_{2},\cdots x_{N}\;\right\}}^{T}\;\min M\left(X\right)=\sum_{e=1}^{N}x_{e}\rho V_{0}\;s.t.\;\mathrm{KU}=\sum_{e=1}^{N}x_{e}^{p}k_{0}u_{e}=F\;\left|\nu -\nu_{0}\right|\leq \varepsilon \;f_{\mathrm{vol}}^{\prime }\leq V\left(X\right)/V_{0}\leq f_{\mathrm{vol}}^{\prime \prime }\;0<x_{\min}\leq x_{e}\leq x_{\max}\leq 1\;e=1,\cdots ,N,$$

where $X={\left\{x_{1},x_{2},\cdots x_{N}\;\right\}}^{T}$ is the vector of relative density. $x_{\max}$ and $x_{\min}$ are the upper and lower limits of the design variables, respectively (non-zero to avoid singularity). *M* is the total mass of functional element. F and U are the vectors of global loading and displacement. $u_{e}$ is the displacement vector of each mesh element corresponding to the design variables. $V\left(X\right)=\sum_{e=1}^{N}x_{e}V_{e0}$ is the total structural volume in optimization progress, where $V_{e0}$ is the initial volume when the relative density of the *e*-th finite element mesh is 1. $V_{0}$ is the initial total volume when the relative density of the design domain area is 1. $f_{\mathrm{vol}}^{\prime }$ and $f_{\mathrm{vol}}^{\prime \prime }$ are the lower and upper limit volume fractions in the design domain. $\left|\nu -\nu_{0}\right|\leq \varepsilon$ is the constraint of PR (ε=0.01), where ν and $\nu_{0}$ represent the PR in optimization iteration and the specified design requirements, respectively.

### 3.3. Optimization Solutions of Lightweight Metamaterials

On one hand, metamaterials should have lightweight properties; thus, the upper limit of the volume fraction is set to 20% to avoid the metamaterials being too heavy after optimization. On the other hand, the usage of materials should not be lower than the lower limit of 20% to guarantee the stability of the optimized metamaterials. That is, the upper and lower limits of materials consumption are set as $0.1\leq V\left(X\right)/V_{0}\leq 0.2$.

**Figure 3:**
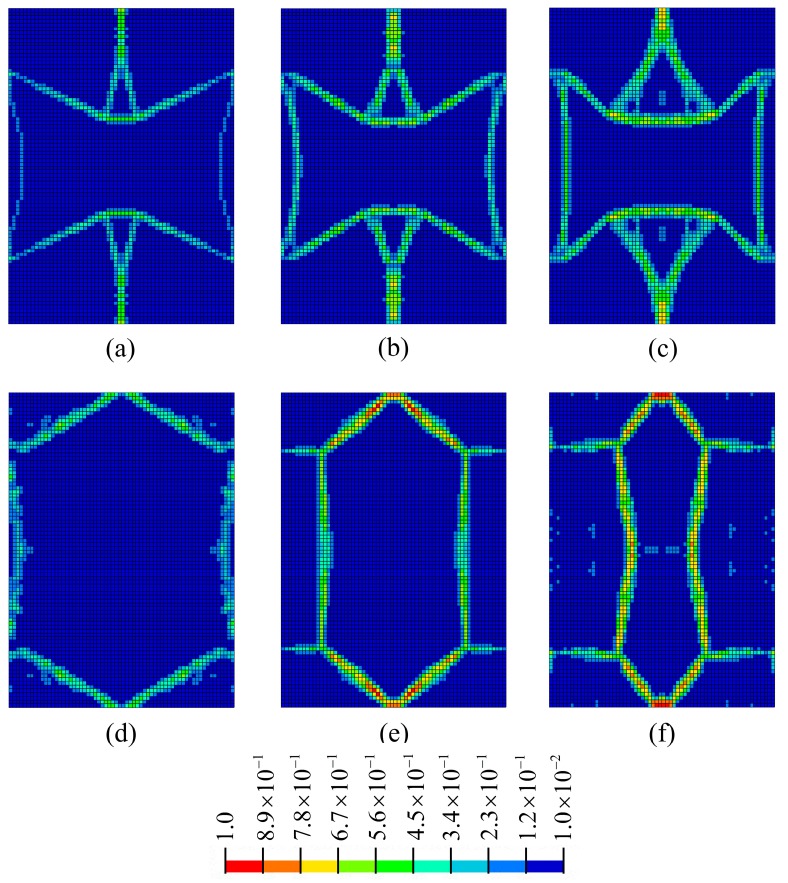
Optimal configurations of functional element corresponding to various Poisson’s ratio (PR) values: (**a**) ν=−0.3; (**b**) ν=−0.6; (**c**) ν=−1.0; (**d**) ν=+0.3; (**e**) ν=+0.6; (**f**) ν=+1.0.

When the PR values are taken as $\nu_{0}=-0.3,-0.6,-1.0,+0.3,+0.6\;\mathrm{and}+1.0$, the corresponding optimal topology configurations are shown in Figure 3 (the solutions are derived in commercial software HyperWorks, and the value of penalization power is 1.5); relative density values are represented by various colors as shown in Figure 3. It is found that the optimized configurations possess clear material distribution paths, and the optimized values of PR are close to the specified design requirements (Section 3.4 verifies the error rate of PR values between the optimized configurations and design requirements).

### 3.4. Extraction of the Optimal Configurations 

At the end of the optimization iteration, the wall thicknesses of the functional elements are not uniform (as shown in Figure 3), which is not convenient to manufacturing. Gibson [32] verified that wall thickness has a negligible effect on mechanical properties when the wall thickness is much smaller than the cell size. Thus, this work ignores the performance changes caused by inconsistent wall thickness, and the optimal configurations are extracted as the functional elements with equal wall thickness *t* = 1 mm.

**Figure 4:**
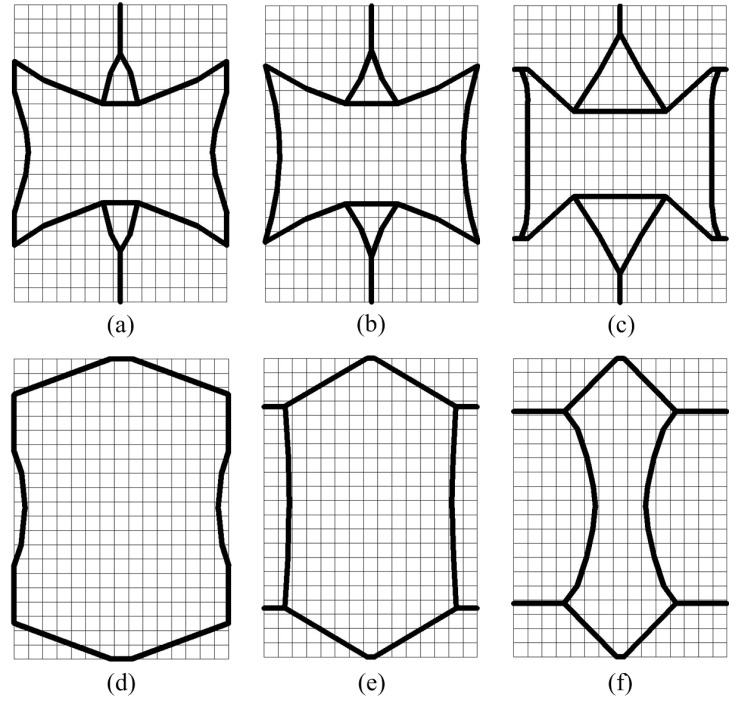
Extraction of optimal configurations corresponding to various PR values: (**a**) ν=−0.3; (**b**) ν=−0.6; (**c**) ν=−1.0; (**d**) ν=+0.3; (**e**) ν=+0.6; (**f**) ν=+1.0.

Considering the irregularity of configurations and the inconvenience of dimensioning, uniformly spaced grids are drawn to measure the dimensions. As shown in Figure 4, the complete area covered by the grids is 42 mm × 30 mm, and the size of each grid is 2 mm × 2 mm.

Due to the inevitable errors in the extraction process of the optimized configurations, we need to reanalyze the extracted configurations of functional elements. Table 1 summarizes the relative errors of Poisson’s ratio between the extracted configurations and design requirements. It can be seen that the PRs of the extracted functional elements are in good agreement with the design requirements.

It should be noted that the metamaterials designed in this work are anisotropic materials because the PR in each direction is different. The major and minor Poisson’s ratios are represented by $\nu =\nu_{ZX}=PRZX$ and $\nu_{XZ}=NUZX$, respectively; the Poisson’s ratio, ν, in this work refers to the major Poisson’s ratio, $\nu_{ZX}=PRZX$.

Ideally, Poisson’s ratio is an inherent property of materials, and does not change with changes in the form, size, etc. of the structure. In this work, we studied the relationship between the PR of a single functional element and the PR effects of periodically arranged metamaterials.

**Figure 5:**
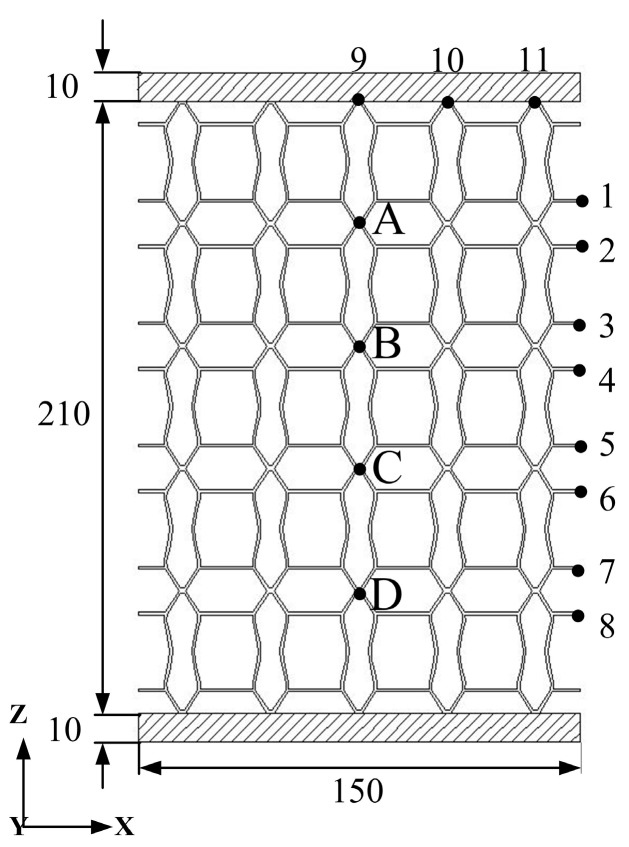
Metamaterials and the location of measuring points with ν=+1.0 (unit: mm).

**Figure 6:**
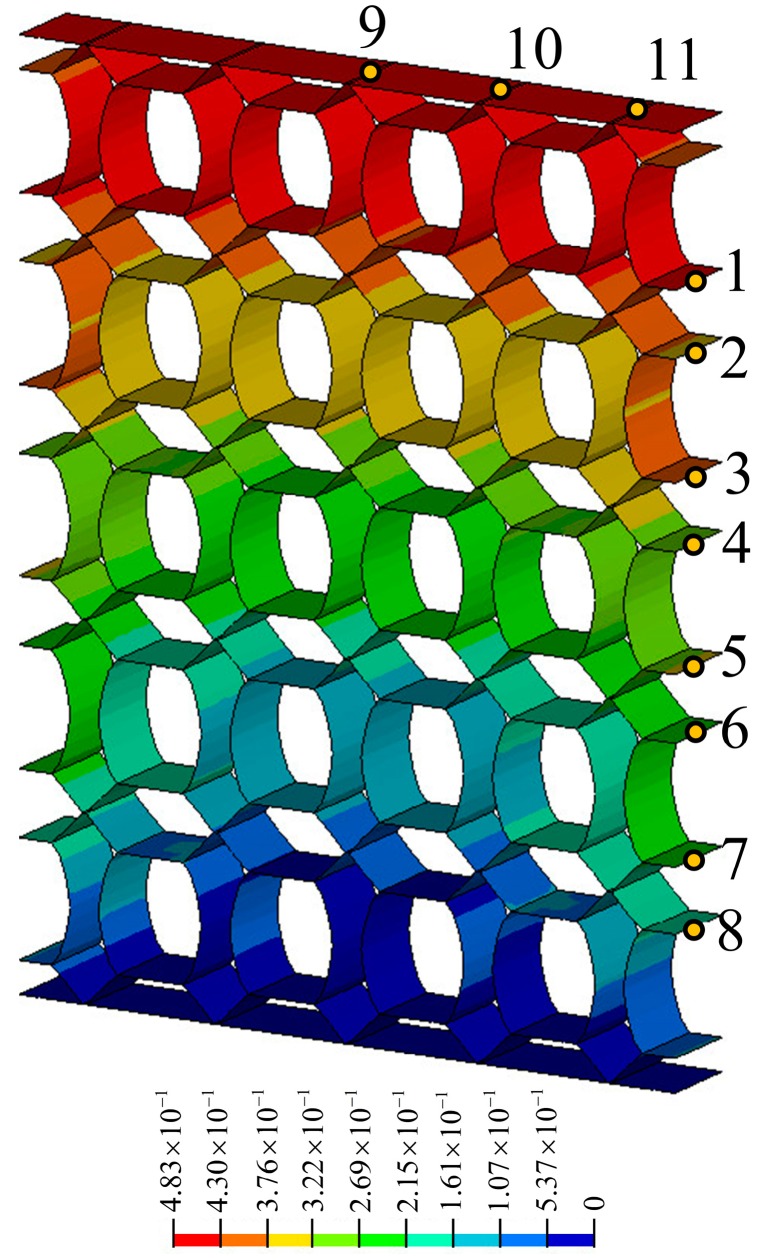
Displacement contour plot of metamaterials with ν=+1.0.

Figure 5 shows the dimensions and measuring points of the metamaterials with ν=+1.0, and Figure 6 shows the static analysis of the metamaterials of Figure 5. The measuring points 1–8 are used to describe the displacement in the X-direction; the average displacement of the eight measuring points is 0.180 mm, which means that the expansion in the X-direction is 0.360 mm, and the strain corresponding to the expansion is 2.40 × 10^−3^. In addition, the measuring points 9–11 are used to describe the displacement in the Z-direction, and the average displacements of these three points is 0.470 mm. Thus, the tensile in the Z-direction is 0.470 mm, and the strain corresponding to the expansion is 2.24 × 10^−3^. Finally, the macroscopic PR effect of the metamaterials is +1.07, and the error rate between this value and the PR value of the single functional element (ν=+1.0) is 7.0%. Therefore, we conclude that the macroscopic PR effects of the metamaterials structure agrees with the PR of the single functional element.

### 3.5. Metamaterials Formation Based on Periodic Arrangement of Functional Elements

**Figure 7:**
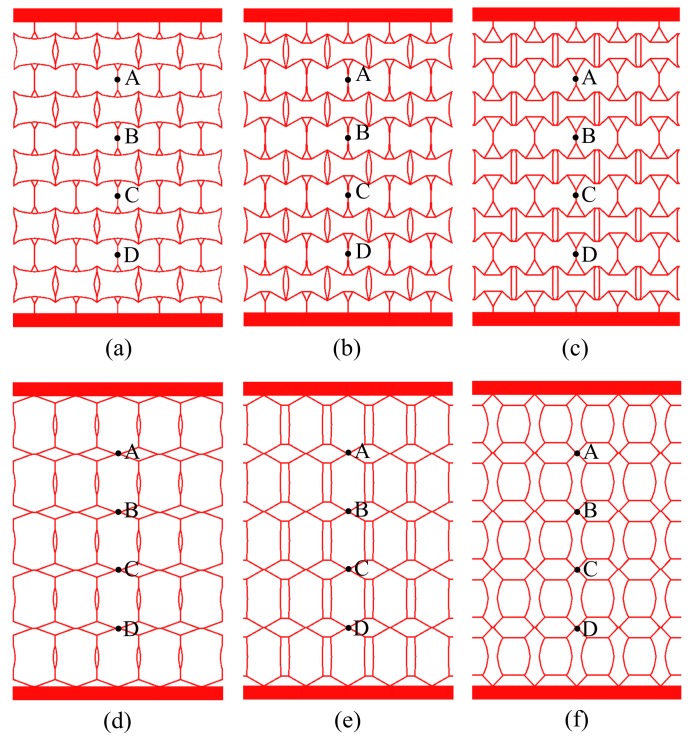
Metamaterials of various PR values: (**a**) ν=−0.3; (**b**) ν=−0.6; (**c**) ν=−1.0; (**d**) ν=+0.3; (**e**) ν=+0.6; (**f**) ν=+1.0.

**Figure 8:**
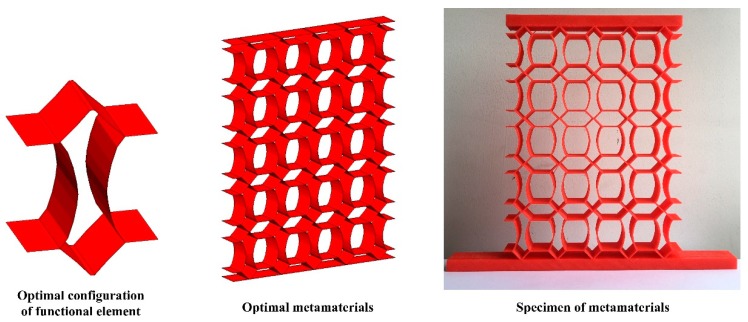
Optimal functional element, metamaterials and prototype with corresponding Poisson’s ratio  ν=+1.0.

Each optimal configuration in Figure 4 is periodically arranged to form the five-layer and five-column metamaterials prototypes (as shown in Figure 7). A concentrated load is applied at the center of the upper faceplate and the lower faceplate is fixed, and the thickness of both faceplates is 10 mm. The outline dimensions are 150 mm × 230 mm, and the depth perpendicular to the page is 20 mm. The prototypes of metamaterials in Figure 7 are fabricated by additive manufacturing, and the materials’ properties are consistent with the parameters in Section 2.2. Figure 8 shows the functional element, metamaterials and prototypes with ν=+1.0.

In this work, analysis is of thin plates with equal thickness, which only receive surface force parallel to the plate surface and do not vary along the thickness at the edge of the plate. The physical force is also parallel to the plate surface and does not vary along the thickness. Therefore, the prototypes shown in Figure 7 can be classified into 2D prototypes.

## 4. Evaluation Method of Static Properties of Metamaterials 

Applications of materials require not only high strength and high stiffness, but also light weight. For low-density materials, strength and stiffness are not prominent, but the ratios of strength and stiffness to density are high, as in magnesium alloys, composites, and honeycombs [42]. To facilitate the comparison of deformation resistance of various metamaterials, the concept of specific stiffness is introduced, which is defined as the ratio of tensile stiffness to the density of materials. The expression of the specific stiffness, κ is as follows:

(8) $$\kappa =ES/\lambda \;\left(N\cdot m^{2}/\mathrm{kg}\;\right)$$

where ES=P/μ is the equivalent stiffness of metamaterials, *P* is the tensile load acting on metamaterials, and μ is the corresponding deformation. $\lambda =W/V_{s}$ is the equivalent density of metamaterials, *W* and $V_{s}$ are the mass and outline volume (overall dimensions), respectively.

**Figure 9:**
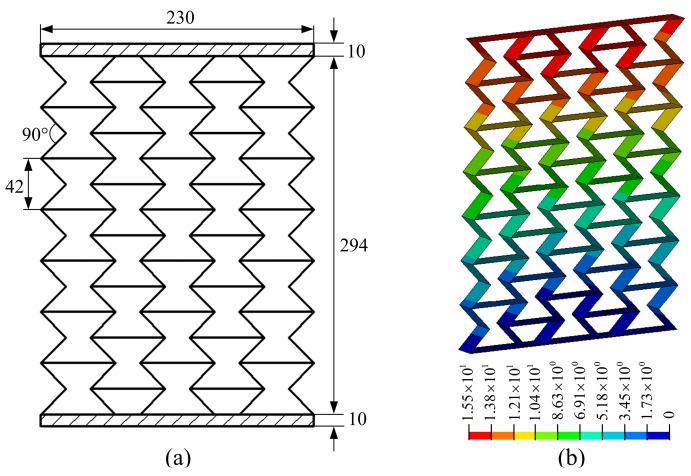
Honeycomb materials in the work by Zhang et al. [43]: (**a**) structural size parameters (unit: mm); (**b**) displacement contour.

**Figure 10:**
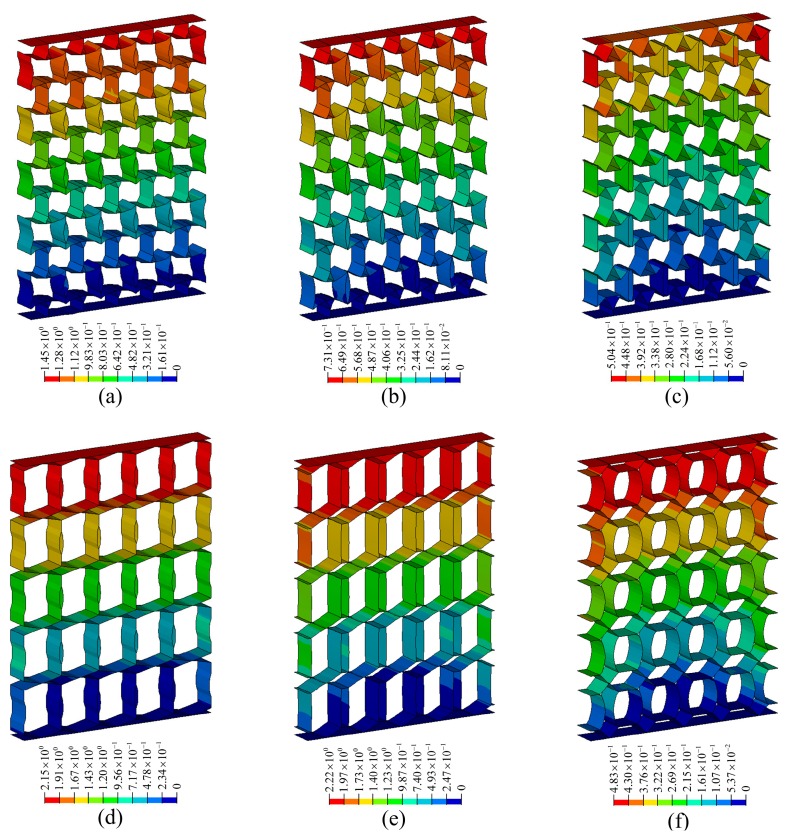
Displacement contours of metamaterials corresponding to various PR values: (**a**) ν=−0.3; (**b**) ν=−0.6; (**c**) ν=−1.0; (**d**) ν=+0.3; (**e**) ν=+0.6; (**f**) ν=+1.0.

Although the load, structure outline dimensions, material properties, and structural form of each metamaterial are not uniform, it is convenient to compare the deformation resistance among various metamaterials by calculating the specific stiffness. In Figure 6, a load of 1 N is applied to each node on the upper faceplate, and the corresponding total load, *P* is shown in Table 2. (For example, if there are 100 nodes in the upper faceplate, the total load, *P* will be 100 N). In Figure 9 and Figure 10, the displacement contours of six metamaterials prototypes and honeycomb are obtained by static analysis, and the displacements at the upper faceplates are extracted and averaged to represent the Z-direction deformation, μ. The values of μ, *W* and $V_{s}$ are summarized in Table 2.

It should be noted that although the magnitude of the load applied to each metamaterial in Figure 7 is not uniform, the effect of load magnitude on the deformation resistance performance of the metamaterials can be avoided by the specific stiffness method. This is due to the fact that the equivalent stiffness, ES represents the inherent property of deformation resistance, and the value of ES is not affected by external loads during the elastic deformation state of the metamaterials.

**Figure 11:**
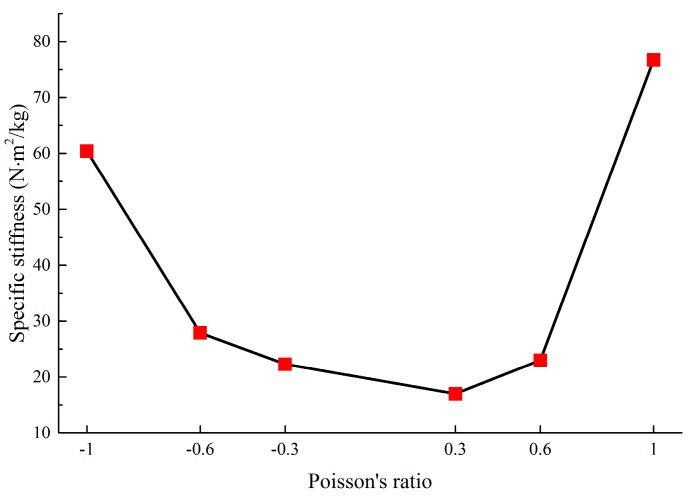
Specific stiffness (deformation resistance) of metamaterials with various PR.

By comparing with honeycomb metamaterials by Zhang et al. [43], the stiffness properties of the novel metamaterials are demonstrated. Comparison in Table 2 shows that the novel metamaterials exhibit higher specific stiffness values than honeycomb. Specifically, the ratios of the specific stiffness of the novel metamaterials to that of the honeycomb are 5.7, 6.6, 13.1, 4.8, 6.9 and 22.0, respectively, which means that the optimized metamaterials have advantages in terms of deformation resistance. In addition, further analysis in Figure 11 shows that the deformation resistance of the novel metamaterials increases with increasing absolute values of Poisson’s ratio.

## 5. Evaluation Method of Vibration Reduction of Metamaterials

Vibration performance of continuous systems is of great practical importance, as vibration with constraints implies cyclic stresses and inevitable fatigue damage. Compared with traditional systems, metamaterials decrease the propagation of vibrations more efficiently and exhibit higher dynamic stiffness [44]. Numerical analyses for the vibration performance of the metamaterials are carried out in this section.

### 5.1. Frequency Response Analysis

The commercial software of HyperWorks is used to build the finite element model of metamaterials and applied to calculate the modal and frequency responses. The lower faceplates are fixed while an axial excitation load, *P* = 10 N is applied at the center of the upper faceplates with sweep bandwidth of 5–200 Hz, and critical damping coefficient of 1%. It should be noted that the frequency range of the honeycomb vibration damping performance by Zhang et al. [43] is 10–100 Hz. In order to compare with the honeycomb research, the frequency range of this work is chosen to be 5–200 Hz.

The Lanczos method [45] is used to calculate the first 50 order modes of metamaterials; Table 3 shows the vertical first-order natural frequency. The dynamic vibration behaviors of the metamaterials are evaluated by the frequency response of measuring points A–D in Figure 7.

### 5.2. Vibration Reduction Performance

The vibration evaluation indicators include vibration level difference, power flow, insertion loss, etc. Among these, the vibration level difference is more convenient and reasonable than other indicators in terms of measurement or evaluation effectiveness, and is more commonly used in applications.

The concept of the vibration level difference (VLD) [46] is used to measure the attenuation or suppression efficiency of the vibration isolation device against the vibration of the vibration source. The larger the vibration level difference, the better the vibration reduction effect of the vibration isolation device. Therefore, the acceleration vibration level difference is used to evaluate the vibration reduction performance of metamaterials.

In this work, the acceleration vibration level difference of the functional elements in the central area is used to evaluate the vibration behavior of the metamaterials. The three functional elements to be measured correspond to the measuring points A–B, B–C, and C–D. For example, *VLD*_A–B_ means the *VLD* between measuring points A and B. Similarly, *VLD*_B–C_ and *VLD*_C–D_ mean the *VLD* between the points B–C and C–D, respectively. The *VLD* values between measuring points *i-j* can be expressed as:

(9) $$VLD_{i–j}=20\;\log_{10}\left(\frac{a_{i}}{a_{j}}\right)\;\left(\mathrm{dB}\right)$$

where $a_{i}$ and $a_{j}$ are the acceleration amplitudes of the measuring points *i* and *j*, respectively.

**Figure 12:**
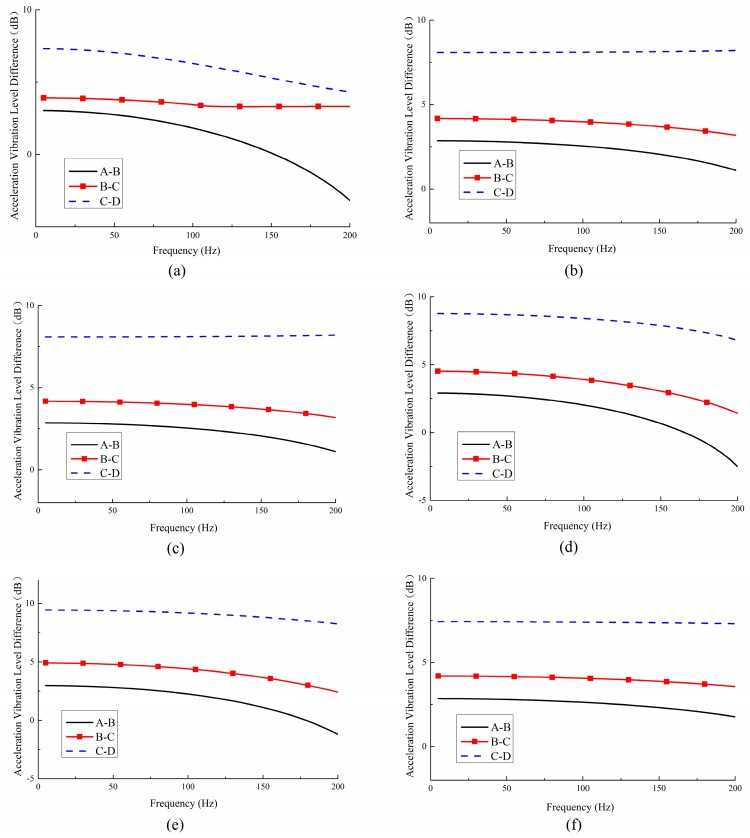
The vibration level difference (VLD) between measuring points A–B, B–C, and C–D corresponding to metamaterials with various PR values: (**a**) ν=−0.3; (**b**) ν=−0.6; (**c**) ν=−1.0; (**d**) ν=+0.3; (**e**) ν=+0.6; (**f**) ν=+1.0.

Figure 12 plots the VLD curves between each two measuring points with a given Poisson’s ratio, which depicts that for a metamaterial with a certain PR value, the closer the location of measuring points to the fixed lower faceplates, the higher the VLD values. Part of the explanation is that the presence of boundary effects results in a larger VLD value between two points closer to the fixed constrained position.

**Figure 13:**
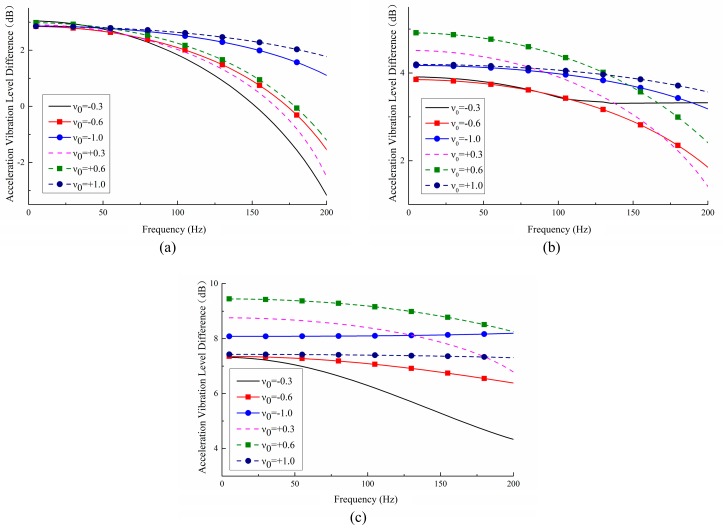
Influence of PR values on the VLD between measuring points: (**a**) A and B; (**b**) B and C; (**c**) C and D.

An in-depth analysis is performed to show the influence of Poisson’s ratio and vibration reduction performance (as shown in Figure 13). Under the premise of selecting similar measuring points, the vibration reduction performance of the six metamaterials is compared and summarized. It can be found that the vibration reduction of positive PR metamaterials is better than that of the negative ones.

The VLDs between A–B, B–C, and C–D measuring points are averaged to comprehensively describe the vibration reduction characteristics of the six metamaterials, which can be expressed as

(10) $$\bar{VLD}=\;({\mathrm{VLD}}_{A\text{–}B}\;+\;{\mathrm{VLD}}_{B\text{–}C}\;+\;{\mathrm{VLD}}_{C\text{–}D})/3$$

**Figure 14:**
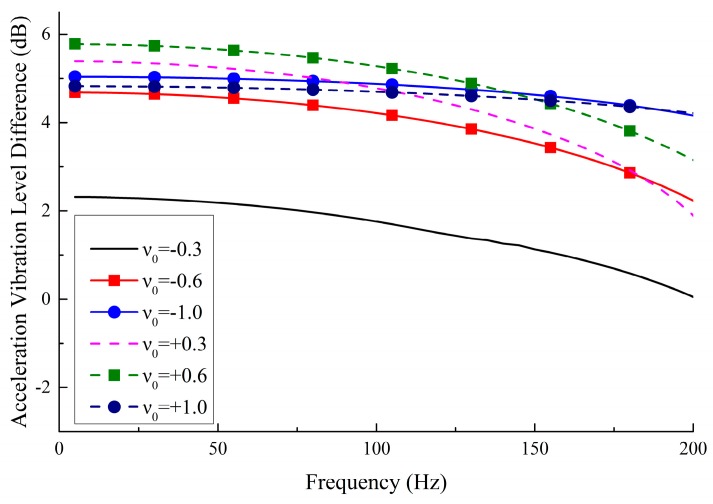
Average vibration reduction performance of the metamaterials with various PR.

As shown in Figure 14, averaging the three VLD curves of each graph in Figure 12 yields the $\bar{VLD}$ of the metamaterials with various PR. It shows that all $\bar{VLD}$ except for the metamaterials with ν=−0.3 are close to 5 dB, which means the amplitude of the vibration is reduced by 43% after the vibration passes through the metamaterials. From the data in Figure 12 and Figure 13, it can be concluded that the metamaterials designed in this work can effectively reduce structural vibration.

### 5.3. Relationship between PR and Vibration Reduction Performance

In order to express the vibration reduction performance more intuitively in the entire frequency band (5–200 Hz), the data in Figure 14 are further analyzed, and the curves of $\bar{VLD}$ are calculated as synthesized value ${\bar{VLD}}^{\mathrm{all}}$ by:

(11) $${\bar{VLD}}^{\mathrm{all}}=\frac{1}{S}\sum_{i=1}^{S}{\bar{VLD}}_{fi}$$

where, $f_{i}=5+5i\;\mathrm{Hz}$, *i* = 1, 2, ⋯, *S*, and *S* equals 39 when the sweep frequency ranges from 5 to 200 Hz. ${\bar{VLD}}_{f_{i}}$ represents the amplitude of $\bar{VLD}$ at the specified frequency $f_{i}$.

**Figure 15:**
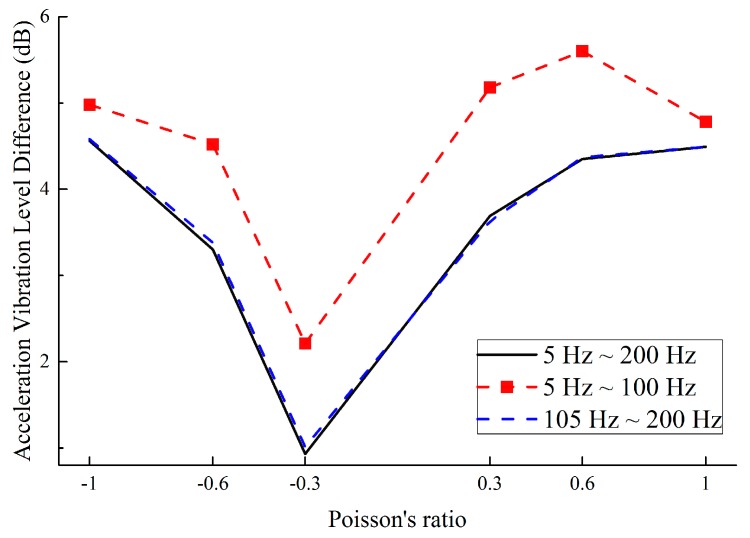
Synthesized vibration reduction performance with various PR in three frequency bands: 5–200 Hz, 5–100 Hz and 105–200 Hz.

Figure 15 summarizes the synthesized values in three frequency bands of 5–100 Hz, 105–200 Hz and 5–200 Hz. In the range of 5–100 Hz, the vibration reduction is 0.5 dB higher than that of other frequency bands, which means the novel designed metamaterials exhibit better vibration reduction at low-frequency. In addition, as the absolute value of the PR increases, the vibration reduction performance is also improved.

**Figure 16:**
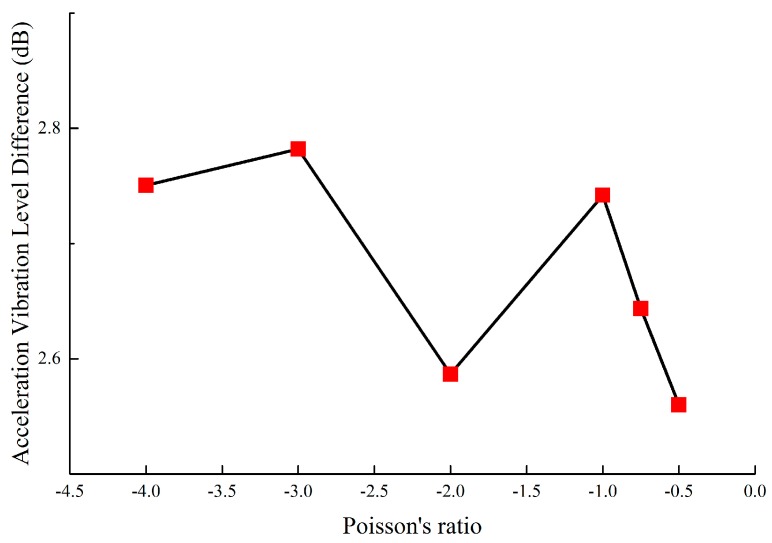
Vibration reduction performance of honeycomb.

Furthermore, this work compares the vibration reduction performance of the novel designed metamaterials and honeycomb by Grima et al. [44]. The external dimensions are shown in Figure 8 and the wall thickness is 1 mm. The same analytical strategy Grima et al. [44] was introduced into this work to study the influence of PR and wall thickness on the vibration reduction performance of honeycomb. Figure 16 shows the vibration reduction performance of honeycomb, which is below 2.8 dB; however, the vibration reduction of the novel lightweight designed metamaterials is at least 4.5 dB, indicating a 60% improvement in vibration reduction performance. Moreover, for the novel metamaterials and honeycomb with ν=−1.0, the vibration reduction performance is 2.7 dB and 4.6 dB, respectively, which indicates the performance of the novel metamaterials is an improvement of 70% compared to honeycomb.

## 6. Lightweight Effects on Deformation Resistance and Vibration Reduction

In this section, the concepts of specific stiffness and specific vibration reduction are defined to evaluate the lightweight effects on the deformation resistance and vibration reduction, respectively.

Section 4 analyzed the specific stiffness (κ) to describe the anti-deformation ability under the premise of a lightweight material, and the influence of each Poisson’s ratio on the deformation resistance of lightweight metamaterials is summarized. The analysis (in Figure 11 and Table 2) shows that compared with honeycomb materials, the novel designed lightweight metamaterials exhibit excellent lightweight and deformation resistance.

Next, we define a concept of specific vibration reduction (ς) to describe the vibration reduction performance under the premise of a lightweight material, which can be expressed as follows:

(12) $$\varsigma ={\bar{VLD}}^{\mathrm{all}}/W\;\left(\mathrm{dB}/\mathrm{kg}\right)$$

where mass *W* is shown in Table 4 (excluding the mass of upper and lower faceplates of the prototypes), and the frequency band of the synthesized vibration level difference ${\bar{VLD}}^{\mathrm{all}}$ is 5–200 Hz (as shown in Figure 15). The values of specific vibration reduction, ς, are calculated (in Table 4) and normalized to observe the influence of each PR on the vibration reduction performance with equal mass.

**Figure 17:**
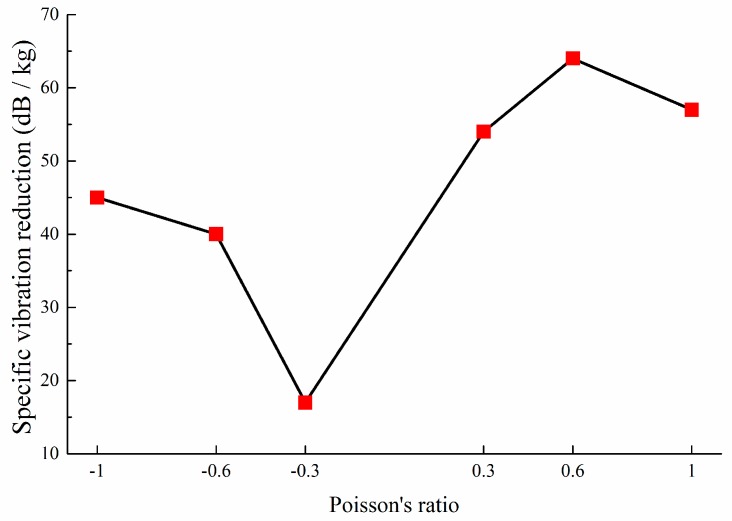
Lightweight effect on vibration reduction performance, corresponding to various PR metamaterials.

From Table 4, under the condition of equal materials consumption, the lightweight metamaterial with ν=+0.6 exhibits significant vibration reduction performance. Figure 17 shows that the performance of metamaterials with positive PR are superior to that of the negative PR metamaterials, which means that the positive PR metamaterials exhibit better lightweight properties in vibration reduction.

## 7. Conclusions

Based on the FETO method by Qin et al. [24], this work systematically studies the design method of lightweight metamaterials. In addition, mechanical properties for the optimal designed metamaterials are analyzed by numerical simulations. The detailed conclusions are as follows:

- This work not only provides several new configurations of metamaterials, but the core innovation is to propose a design method of lightweight metamaterials by establishing a mathematical optimization model. Moreover, the design method enables the design of lightweight metamaterials with a specified Poisson’s ratio value.
- Comparison with honeycomb materials shows that the designed novel metamaterials exhibits outstanding deformation resistance and vibration reduction performance.
- The concepts of specific stiffness, κ, and specific vibration reduction, ς, are defined to evaluate the lightweight effects of novel metamaterials on deformation resistance and vibration reduction, respectively.

## Figures and Tables

**Table 1:** Relative errors of Poisson’s ratio between the extracted configurations and design requirements ($\nu =\nu_{ZX}=PRZX$).

**Design Requirements**	ν=−0.3	ν=−0.6	ν=−1.0	ν=+0.3	ν=+0.6	ν=+1.0
**After Extraction**	$\nu^{\prime }=-0.316$	$\nu^{\prime }=-0.583$	$\nu^{\prime }=-1.027$	$\nu^{\prime }=+0.303$	$\nu^{\prime }=+0.578$	$\nu^{\prime }=+1.08$
**Relative Error Ratio**	+5.3%	−2.8%	+2.7%	+1.0%	−3.7%	+8.0%

**Table 2:** In-plane specific stiffness of metamaterials.

Metamaterials	*P*/*N*	μ/mm	$ES/\left(N\cdot {\mathrm{mm}}^{-1}\right)$	*W*/kg	$V_{s}/{\mathrm{mm}}^{3}$	$\kappa /\left(N\cdot m^{2}/\mathrm{kg}\right)$
Honeycomb with ν=−0.5	33	15.07	2.2	1.092 × 10^−1^	294 × 230 × 20	27.2
ν=−0.3	31	1.39	22.3	9.13 × 10^−2^	210 × 150 × 20	153.9
ν=−0.6	19	0.68	27.9	9.81 × 10^−2^	210 × 150 × 20	179.4
ν=−1.0	25	0.41	60.4	1.071 × 10^−1^	210 × 150 × 20	355.3
ν=+0.3	36	2.12	17.0	8.18 × 10^−2^	210 × 150 × 20	130.9
ν=+0.6	50	2.17	23.0	7.77 × 10^−2^	210 × 150 × 20	186.7
ν=+1.0	36	0.47	76.7	8.06 × 10^−2^	210 × 150 × 20	599.5

**Table 3:** Vertical first-order natural frequency of metamaterials corresponding to various PR.

**Metamaterials with Various PR**	ν=−0.3	ν=−0.6	ν=−1.0	ν=+0.3	ν=+0.6	ν=+1.0
**Natural Frequency**	89.5 Hz	97.5 Hz	136.2 Hz	78.1 Hz	90.9 Hz	163.1 Hz

**Table 4:** Lightweight effect on vibration reduction performance.

**Poisson’s Ratio**	ν=−0.3	ν=−0.6	ν=−1.0	ν=+0.3	ν=+0.6	ν=+1.0
**Mass (g)**	91.3	98.1	107.1	81.8	77.7	80.6
${\bar{VLD}}^{\mathrm{all}}$ **(dB)**	1.56	3.95	4.78	4.40	4.99	4.63
**Specific Vibration Reduction** ς **(dB/kg)**	17	40	45	54	64	57
**Normalized** ς	0.27	0.63	0.70	0.84	1.00	0.90
